# T.E.A. Study: three-day ertapenem versus three-day Ampicillin-Sulbactam

**DOI:** 10.1186/1471-230X-13-76

**Published:** 2013-04-30

**Authors:** Fausto Catena, Carlo Vallicelli, Luca Ansaloni, Massimo Sartelli, Salomone Di Saverio, Riccardo Schiavina, Eddi Pasqualini, Annalisa Amaduzzi, Federico Coccolini, Michele Cucchi, Daniel Lazzareschi, Gian Luca Baiocchi, Antonio D Pinna

**Affiliations:** 1 Emergency Surgery Department, Parma University Hospital, Parma, Italy; 2 General and Transplant Surgery Department, St. Orsola-Malpighi University Hospital, Bologna, Italy; 3 General and Emergency Surgery Department, Ospedali Riuniti of Bergamo, Bergamo, Italy; 4 General Surgery Department, Hospital of Macerata, Macerata, Italy; 5 General and Trauma Surgery Department, Maggiore Hospital, Bologna, Italy; 6 Unit of Urology, St. Orsola-Malpighi University Hospital, Bologna, Italy; 7 Department of Integrative Biology, University of California, Berkeley, USA; 8 Department of Medical and Surgical Sciences, Surgical Clinic, Brescia University, Brescia, Italy

## Abstract

**Background:**

Intra-abdominal infections are one of the most common infections encountered by a general surgeon. However, despite this prevalence, standardized guidelines outlining the proper use of antibiotic therapy are poorly defined due to a lack of clinical trials investigating the ideal duration of antibiotic treatment. The aim of this study is to compare the efficacy and safety of a three-day treatment regimen of Ampicillin-Sulbactam to that of a three-day regimen of Ertapenem in patients with localized peritonitis ranging from mild to moderate severity.

**Methods:**

This study is a prospective, multi-center, randomized investigation performed in the Department of General, Emergency, and Transplant Surgery of St. Orsola-Malpighi University Hospital in Bologna, Italy. Discrete data were analyzed using the Chi-squared and Fisher exact tests. Differences between the two study groups were considered statistically significant for p-values less than 0.05.

**Results:**

71 patients were treated with Ertapenem and 71 patients were treated with Ampicillin-Sulbactam. The two groups were comparable in terms of age and gender as well as the site of abdominal infection. Post-operative infection was identified in 12 patients: 10 with wound infections and 2 with intra-abdominal infections. In the Ertapenem group, 69 of the 71 patients (97%) were treated successfully, while the therapy failed in 2 cases (3%). Therapy failures were more frequent in the Unasyn group, amounting to 10 of 71 cases (p = 0.03).

**Conclusion:**

According to these preliminary findings, the authors conclude that a three-day Ertapenem treatment regimen is the most effective antibiotic therapy for patients with localized intra-abdominal infections ranging from mild to moderate severity.

**Trial registration:**

Trial registration: ClinicalTrials.gov: NCT00630513

## Background

Intra-abdominal infections are one of the most common infections encountered by a general surgeon. These infections are difficult to diagnose in their early stages and effective treatment is equally challenging. Ertapenem and Ampicillin-Sulbactam are both recommended as single agent treatments to address localized peritonitis by the SIS (Surgical Infections Society) and the IDSA (Infection Diseases Society of America). According to these guidelines, “an antimicrobial therapy for established infections should be continued until resolution of clinical signs of infection occurs, including normalization of temperature and white blood cell count and return to gastrointestinal function” [1]. The ambiguity of these guidelines is the result of a distinct lack of clinical trials investigating the optimal duration of antibiotic therapy. Consequently, in most trials involving antibiotic therapy, an arbitrarily fixed period ranging from 5 to 14 days is used for all patients with intra-abdominal infections, irrespective of the severity of peritonitis. Most patients enrolled in trials involving intra-abdominal infections present with mild peritonitis and acute appendicitis [2]. Furthermore, most cases feature localized infections or simple contamination. These patients often receive superfluous antibiotic treatment. A meta-analysis of twenty-eight studies investigating the duration of antibiotic treatment in cases of advanced pediatric appendicitis demonstrated that limiting the duration of antibiotic treatment to three days was not associated with a higher rate of abdominal abscesses or wound infection [3]. A randomized study demonstrated that shorter duration of Ertapenem therapy is as effective as a 5-day treatment regimen in patients with mild to moderate peritonitis [4]. Given that overuse of antibiotics increases treatment costs, reduces clinical efficacy, and spurs the emergence of antibiotic-resistant pathogens and bacterial strains [5], a conservative shift in antibiotic methodology is of utmost importance.

The aim of this study is to compare the efficacy and safety of a three-day treatment regimen of Ampicillin-Sulbactam to that of a three-day regimen of Ertapenem in patients with localized peritonitis ranging from mild to moderate severity. All patients had a localized peritonitis. The Basoli study demonstrated that, in patients with localized community-acquired intraabdominal infection, a 3-day course of ertapenem had the same clinical and bacteriological efficacy as a standard duration [4].

Ertapenem is a long-term acting parenteral Group I carbapenem, which acts in vitro against most aerobic and anaerobic bacteria associated with community-acquired infections [6,7]. Although it is not active against Pseudomonas Aeruginosa or Enterococci, prevention of such strains is not routinely required for intra-abdominal infections [8,9]. Ampicillin-Sulbactam is widely used in the treatment of intra-abdominal infections. The daily dosage is 3 g × 3 compared to Ertapenem, which is administered as a 1-g single dose. It should be noted that, in Italy, 1 g of Ertapenem costs 36 euros while 1 g of Ampicillin-Sulbactam costs only 0.23 euros.

## Methods

This study is a prospective, randomized investigation performed in the Department of General, Emergency, and Transplant Surgery at St. Orsola-Malpighi University Hospital in Bologna, Italy, and was conducted by all surgeons willing to participate in the study. Patients were enrolled in the study during a two-year period, January 2008 to March 2010. The study was designed and conducted in compliance with the regulations outlined in “Good Clinical Practice” methodology. The efficacy of a 3-day treatment regimen of Ampicillin-Sulbactam (AS 3 g × 3/day administered intravenously) was compared to a 3-day regimen of Ertapenem (1g/day administered intravenously) in the treatment of patients with localized peritonitis followed by blind evaluation of pre-established efficacy-based endpoints. Evaluation of success or failure is blindly assessed by designated third party administrators who are unaware of the treatments assigned to patients [10].

### Randomization

Randomization was achieved using a computer-generated assignment system; the results were sealed in numbered envelopes. The patient signed a document verifying informed consent at which time the attending surgeon disclosed the contents sealed within the assigned envelope to determine the patient’s randomly assigned treatment group. The attending surgeon then recorded the patient name and envelope number.

### Statistical analysis

Statistical analysis was carried out using Epi Info 2000, Version 1.1 software package. Researchers used the continuous numerical data to perform an analysis of variance (ANOVA), given that this method can discriminate between two continuous populations. Discrete data were analyzed using the Chi-squared or Fisher exact tests, as appropriate. Differences between the two study groups were considered statistically significant for p-values less than 0.05.

### Inclusion and exclusion criteria

Inclusion criteria include the following: adulthood (> 18 years of age), surgical intervention within 24 hours of diagnosis, localized intra-abdominal infection, acute appendicitis, acute perforated diverticulitis (perforated diverticulitis without fluid collection addressed surgically by means of primary anastomosis), acute cholecystitis, acute gastric and duodenal perforation (> 24 hours), traumatic perforation of the intestine (>12 hours), secondary peritonitis due to perforated viscera, and intra-abdominal abscesses.

Exclusion criteria include the following: traumatic bowel perforation requiring surgery within 12 hours, perforation of gastrointestinal ulcer requiring surgery within 24 hours, other intra-abdominal diseases in which the primary etiology was unlikely to be infectious, lactation or pregnancy, rapidly progressive or terminal illness, history or presence of severe hepatic or renal disease, concomitant infections that would interfere with the evaluation of responses to the study antibiotics, and history of allergy, hypersensitivity, or any other severe reaction to study antibiotics [10].

Upon patient enrolment, the severity of disease was evaluated by means of an APACHE II score prior to the operation.

Diagnosis was based on the patient’s clinical symptoms, laboratory tests, and CT imaging: thereafter it was confirmed by microbial cultures and other intra-operative findings.

Upon enrolment, all patients underwent physical examinations and laboratory tests, which were repeated identically on the third day of the treatment regimen, at the end of the treatment cycle following the resolution of pathology, and during the post-treatment follow-up analysis.

Preoperative percutaneous drainage was not performed, as this procedure is not conducted in emergency settings at our institution.

In all cases, treatment began with a laparoscopic approach and transitioned to open surgery if deemed necessary.

### Antibiotic therapy and data collection

The operation was performed within 24 hours of diagnosis, and antibiotic therapy was initiated in the operating room prior to surgery. The patient was then post-operatively administered a 3-day treatment of either Ertapenem or Ampicillin-Sulbactam depending on the assigned treatment group. The treatment was discontinued in patients demonstrating improved body temperature (<37.8°C), a shift in white blood cell counts into the normal range, and the presence of abdominal sounds by the third day of treatment. Aerobic and anaerobic cultures of intra-operatively retrieved specimens were obtained at baseline and processed in the clinical microbiological laboratory. All microorganisms were isolated, cultured, and tested for in vitro susceptibility to the study antibiotics.

Upon enrolment, all patients underwent a series of physical examinations and laboratory tests. The same examinations were performed on the third day of the treatment regimen, at the end of the treatment cycle following the resolution of pathology, and during the post-treatment follow-up analysis. The clinical outcomes of eligible patients were categorized into three groups: successful (no signs or symptoms of infection and no further need for antimicrobial therapy), failure (indicating no improvement, progression of infection, or death due to infection), and late failure (indicating recurrence between cessation of antibiotic therapy and follow-up). All adverse events that occurred during the course of the study were carefully documented.

### Informed consent

Informed consent was obtained in writing from all enrolled participants (adult patients, >18 years old). In the informed consent document, the patient reviewed all relevant information regarding the study protocol and the confidentiality of personal data. After signing the informed consent document, the patient completed a questionnaire. The patients were permitted to withdraw from the study at any time without any further commitments or obligations.

### Ethical approval and study protocol

The study was approved by the Ethics Committee of St. Orsola-Malpighi University Hospital and the study protocol was registered with ClinicalTrials.gov under the title “University of Bologna Protocol Record T.E.A. Study, T.E.A. Study Three days Ertapenem versus three days Ampicillin-sulbactam”.

### Primary endpoint

The primary endpoint involved comparing the clinical efficacy and failure rate of truncated Ertapenem and Ampicillin-Sulbactam antibiotic regimens in the treatment of localized intra-abdominal infections.

## Results

As demonstrated in Table 1, the two groups of patients were comparable in terms of gender and age criteria. The Ertapenem group consisted of 36 men and 35 women, while the Ampicillin- Sulbactam group consisted of 37 men and 34 women. Mean patient ages were 52.3 and 51 years for the Ertapenem and Ampicillin-Sulbactam groups, respectively. In the Ertapenem group, 35 of 71 patients presented with acute appendicitis compared to 31 cases in the Ampicillin-Sulbactam group. 9 (12.6%) and 10 (14%) patients presented with acute diverticulitis in the Ertapenem and Unasyn groups, respectively. Patients with peptic ulcers were rare in both groups: only 1 case in the Ertapenem group and 2 cases in the Ampicillin-Sulbactam group. There were not major differences between the two groups are in term of acute cholecystitis; this condition accounted for 36.6% of all cases in the Ertapenem group (26 patients) and 39.4% in the Ampicillin-Sulbactam group (28 patients). According to Apache II Scores, 7 patients in the Ertapenem group and 8 patients in the Ampicillin-Sulbactam group exhibited scores greater than 10. All other cases featured scores less than or equal to 10. This demonstrates that the severity of illness typically ranged from mild to severe, as indicated in the inclusion criteria of this study.

**Table 1:** Patients’ characteristics and preoperative- intraoperative findings

**Parameter**	**Ertapenem group**	**Ampicillin- Sulbactam group**	**P**
Male/Female Ratio	36/35	34/37	n.s.
Age	52,3	51	n.s.
Apache II score > 10	7	8	n.s.
Phlegmonous appendicitis	11	10	n.s.
Gangrenous appendicitis	12	11	n.s
Appendicitis with abscess	12	10	n.s
Localized perforated diverticulitis without fluid collections	9	10	n.s
Phlegmonous cholecystitis	9	10	n.s
Gangrenous cholecystitis	9	10	n.s
Perforated cholecystitis with localized collection	8	8	n.s
Perforated gastric ulcer	1	1	n.s
Perforated duodenal ulcer	0	1	n.s
Conversion to open surgery	7	8	n.s

The conversion rate from laparoscopic to open surgery was comparable in both studied groups (7/71 in the Ertapenem group and 8/71 in the Ampicillin-Sulbactam group; p= n.s.).

As reported in Table 2, post-operative infections occurred in 12 patients, 10 presenting with a superficial infection and 2 with a deep intra-abdominal infection. Both deep intra-abdominal infections and 8 of the 10 superficial infections occurred in the Ampicillin-Sulbactam group.

**Table 2:** Outcomes

**Diagnosis**	**Ertapenem group**	**Ampicillin- Sulbactam group**	**p**
Superficial/Deep Infections	2/0	8/2	0.03
Mortality	1 (AMI)	1 (PE)	n.s.
Morbidity	21	19	n.s.
PE	1	1	n.s.
DVT	3	4	n.s.
AMI	1	1	n.s.
Pneumoniae	6	5	n.s.
Prolonged ileus	4	4	n.s.
UTI	3	3	n.s.
TIA	1	0	n.s.
Biliary leaks	2	1	n.s.
Cure/Failure	69/2	51/10	0.03

Other miscellaneous morbidities involved both treatment groups equally, with 21 cases in the Ertapenem group and 19 cases in the Ampicillin-Sulbactam group (p = n.s. Table 2). Each treatment group experienced 1 case of post-operative death.

Patients presenting with biliary leaks underwent conservative treatment successfully.

As reported Table 2, in the Ertapenem group, 69 of 71 patients (97%) were effectively treated, whereas the therapy failed in 2 cases (3%). Therapy failures were more frequent in the Ampicillin-Sulbactam group, amounting to 10 of 71 cases. As such, 61 of 71 patients (86%) underwent successful antibiotic therapy. The difference in effectiveness of the two antibiotic treatments was determined to be statistically significant (p = 0.03).

Superficial infections were treated in an outpatient setting by means of repeated washes and aggressive irrigation, whereas deep infections were treated with CT-guided percutaneous drainage and second-line antibiotics (piperacillin-tazobactam, 18 grams)

In the Ertapenem group, treatment failure occurred in 2 cases and was caused by Enterobacter, Staphylococci, and Acinetobacter (Table 3).

**Table 3:** Pathogens recovered and their susceptibilities to Ertapenem group and Ampicillin- Sulbactam group

	**Ertapenem group**				**Ampicillin Sulbactam group**			
	**appendicitis**		**non appendicitis**		**appendicitis**		**non appendicitis**	
Pathogen	S	R	S	R	S	R	S	R
**Aerobes Gram-positive**								
Staphylococcus coagulase-negative	1							
Staphylococcus haemolyticus	1				2	2	4	
Other staphylococci	2	1	3		2			
Streptococci			6				2	
Enterococcus faecium								3
Enterococcus faecalis					1			
Other enterococci			2				2	
**Aerobes Gram-negative**								
Escherichia coli	36		9		21	3	23	
Enterobacter cloacae				1				
Enterobacter faecalis			2					
Pseudomonas					2	1		
Klebsiella							3	
Other Enterobacter spp	2							2
Proteus							2	
Serratia								2
Citrobacter								
Acinetobacter baumanii		1						
Anaerobes								
Bacteroides fragilis	5		4		3		4	
Clostridium spp	2							
Fusobacterium frigens			2					
Peptostreptococcus					1			

Contrastingly, in the Ampicillin-Sulbactam group, treatment failure occurred in 10 cases and was caused by Enterobacter, Enterococci, Staphylococci Pseudomonas, Serratia and Escherichia Coli (Table 3).

There were no clostridium difficile infections.

## Discussion

The guidelines of the Surgical Infection Society and the Infectious Disease Society of America state that “an antimicrobial therapy for established infections should be continued until resolution of clinical signs of infection occurs, including normalization of temperature and white blood cell count and return to gastrointestinal function” [1]. These indications are limited and non-specific due to a lack of clinical trials investigating the appropriate duration of antibiotic therapy. As a consequence, many studies rely on an arbitrarily determined “default” duration of antibiotic treatment without accounting for the severity of a particular infection. Many patients are therefore over-treated with superfluous antibiotic therapy, which increases therapy costs, triggers adverse side-effects, and spurs the emergence of drug-resistant pathogens. This precarious situation underscores the need for clinical trials investigating the proper duration of antibiotic therapy.

The Word Society of Emergency Surgery (WSES) made an additional contribution to the debate regarding proper antimicrobial drug methodology [11]. The WSES guidelines outline the proper antimicrobial strategies to address biliary, extrabiliary, community-acquired, and hospital-acquired intra-abdominal infections. The authors maintain that arbitrarily determined “default” antibiotic regimens that do not take into account the circumstances of individual cases result in poorer patient outcomes than do scrupulously customized regimens.

The aim of this study is to compare the efficacy and safety of a three-day treatment regimen of Ampicillin-Sulbactam to that of a three-day regimen of Ertapenem in patients with localized peritonitis ranging from mild to moderate severity. The authors define localized peritonitis as an infectious process that extends beyond a singularly affected viscus into the peritoneal space without involving the entire peritoneal cavity. As in many other studies, most patients presented with acute appendicitis.

Morover patients with localized perforated diverticulitis without fluid collection were submitted to surgery and primary anastomosis. We know that it is not the worldwide accepted standard of care for acute diverticulitis and most of the patients should have been treated with antibiotics. We performed this laparoscopic approach also with diagnostic value and with the intention of a one stage emergency treatment. As underlied in literature, the heterogeneity of patients with colonic diverticular disease means that treatment should be tailored on an individual basis [12].

Ertapenem is a long-term acting parenteral Group I carbapenem, which acts in vitro against most aerobic and anaerobic bacteria associated with community-acquired infections. Ampicillin-Sulbactam is a widely used antibiotic in the treatment of intra-abdominal infections.

This study demonstrates that a 3-day regimen of Ertapenem has the same clinical effectiveness as a 3-day regimen of Ampicillin-Sulbactam in treating patients with localized intra-abdominal infections. Clinical resolution was achieved in 97% of patients in the Ertapenem group compared to just 86% of cases in the Ampicillin-Sulbactam group, which represents a statistically significant difference (p = 0.03).

Regarding the proper duration of antibiotic therapy, the authors determined that a 3-day Ertapenem or Ampicillin-Sulbactam regimen is typically sufficient to safely and efficiently achieve clinical resolution of infection. Many clinical parameters and resolution metrics such as body temperature, white blood cell count, and normalization of gastrointestinal function are important factors that must be taken into account when assessing possible discontinuation of antibiotic treatment. From this study’s data one can infer that the risk of treatment failure following suspension of antibiotic treatment is minimal in patients with no clinical evidence of persistent infection.

The study reported 2 cases of death out of 142 enrolled patients (1.4%), equally distributed between the two treatment groups. Morbidity cases involved wound infection (10 patients, 7%) and intra-abdominal infection (2 patients, 1.4%).

The mild to moderate severity of localized peritonitis was evaluated according to APACHE II and MPI scores. Only 15 patients (10.5%) exhibited an APACHE II score greater than 10. The results of this study can only be applied to those patients with localized, community-acquired intra-abdominal infections who demonstrate clinical improvement after three days of antibiotic therapy. The results of this study should not be extrapolated to patients outside of this categorical group. Additional prospective studies investigating the duration of antibiotic regimens should be conducted to confirm these results.

## Conclusion

Reducing the superfluous consumption of antibiotics reduces the prevalence of bacterial drug resistance and lessens healthcare costs. Discontinuing antibiotic therapy after three days of treatment saves valuable hospital resources by conserving antibiotic medications and limiting unnecessary nursing care. Ultimately, a 3-day antibiotic regimen reduces the overall duration of hospitalization. Based on these preliminary results, the authors conclude that a 3-day Ertapenem regimen is the optimum treatment for patients with localized intra-abdominal infections ranging from mild to moderate severity.

## Competing interests

The authors declare no competing interests.

## Authors’ contribution

CF: Contributed as first author, participating in study conception, in analysis and interpretation of data, in manuscript draft and revision, and in final approval of the manuscript. VC, AL, SM, DSS, SR, PE, AA, CF, CM, LVD, BGL, PAD: Participated in manuscript draft, revision, and final approval. All authors read and approved the final manuscript.

## Pre-publication history

The pre-publication history for this paper can be accessed here:

http://www.biomedcentral.com/1471-230X/13/76/prepub
